# Digital mediation, parental self-efficacy and social context: a cross-sectional study on parents in Sweden

**DOI:** 10.1186/s12889-026-27351-y

**Published:** 2026-04-15

**Authors:** Sara Krantz, Nicholas Matarehua Wikiriwhi Kirkvaag, Sara Svanholm, Louise Arvidsson

**Affiliations:** 1 Department of Public Health, University of Skövde, PO Box 408, Högskolevägen 1, Skövde, 541 28 Sweden; 2 Department of Health Sciences, Mid Sweden University, Holmgatan 10, Sundsvall, 851 70 Sweden

**Keywords:** Parental support program, Digital parenting, Digital mediation, Parental self-efficacy, Health promotion

## Abstract

**Background:**

As children’s digital activities begin earlier and grow more complex, parental responsibilities now extend into digital domains. Digital parenting, including digital mediation, ways in which parents communicate and engage with children around digital media use, has become increasingly vital to promote healthy child development. The aim of this study was to investigate the association between parental individual- and social characteristics, including self-efficacy, gender, native language and social networks, and digital mediation strategies among parents of preschool and elementary school children in Sweden.

**Methods:**

A cross-sectional study was conducted in 2024 using baseline data from an evaluation of a universal parenting programme in a small Swedish municipality. The sample included 177 parents of children aged 3–14 years. Data were collected via a digital survey and analyzed using non-parametric tests and logistic regression. Parental mediation strategies were assessed using the Media Parenting Scale for School-Aged children (MEPA-20), and self-efficacy was measured using the validated Tool to measure Parental Self Efficacy (TOPSE).

**Results:**

Higher parental self-efficacy was significantly associated with more frequent use of active (aOR = 1.2, 95% CI 1.1–1.3) and restrictive (aOR = 1.2, 95% CI 1.1–1.2) mediation strategies. A gender difference was identified, with women reporting higher odds of using active mediation compared to men (aOR = 2.6, 95% CI 1.1-6.0). Parents with a non-Swedish native language were more likely to use restrictive (aOR = 2.6, 95% CI 1.2–5.8) and overprotective (aOR = 5.7, 95% CI 2.4–13.6) mediation, independent of self-efficacy. Institutional networks (e.g. schools, health services) were linked to higher odds of both active (aOR = 2.2, 95% CI 1.1–4.3) and overprotective (aOR = 2.6, 95% CI 1.3–5.2) mediation, while personal networks had no significant influence.

**Conclusions:**

Digital parenting is shaped by parental self-efficacy, social context, and institutional support. Findings underscore the importance of culturally responsive public health interventions that enhance parental confidence and promote balanced digital mediation. Institutional actors play a key role in reaching diverse families, supporting equitable digital environments in line with public health and Agenda 2030 goals.

**Supplementary Information:**

The online version contains supplementary material available at 10.1186/s12889-026-27351-y.

## Background

Children across the world are engaging with digital tools at increasingly younger ages [1]. As digitalization accelerates, parenting responsibilities now extend beyond the physical environment to digital contexts [2–4]. Digital parenting is a broad, holistic concept encompassing parents’ digital competence, attitudes toward technology, and access to support—all of which shape how they guide and protect their children online [5, 6].

In Sweden, internet usage among children is high, with daily digital tool use common from middle school onwards [7]. As a result, parents face growing concerns about screen time, and access to parental support is essential for fostering positive parent–child relationships [2]. While many parents recognize benefits of digital media, such as educational opportunities and social connectivity [7], concerns persist regarding excessive screen time and the formation of unhealthy habits [2]. National data show that few children aged 5–8 exceed three hours of daily screen time, but among those aged 9–12, extended social media use is becoming increasingly common [8, 9].

Many parents report a need for greater guidance on how to mediate their children’s digital media use [2]. Digital parenting introduces new challenges, including setting appropriate boundaries while promoting health and development [7, 10]. Through digital competence, parents can foster children’s independence and confidence in navigating online environments [3]. This includes cultivating critical skills such as source evaluation, digital privacy, and information literacy [7]. Strengthening parental digital mediation capacity – through access to information, communication, and support – is a public health priority [11].

### Digital mediation

A central element of digital parenting is digital mediation, defined as the ways in which parents communicate and engage with children around digital media use [12, 13]. This involves managing internet and digital platform use to mitigate risks and enhance positive outcomes [4]. Earlier research focused on television [14, 15] and internet use [16, 17], but has since evolved to encompass broader digital contexts [18, 19], including mobile gaming and communicating platforms where children coordinate activities and build digital networks [18].

Previously three parental mediation strategies have been discussed: active, restrictive, and co-using [12, 14, 20]. Active mediation involves discussing and interpreting media content with the child during or after use; restrictive mediation focuses on rule-setting that limits time, content, or activities; and co-using mediation refers to shared media use (e.g., co-viewing or co-playing), which may occur with or without explicit discussion [14, 20]. These strategies have generally been treated as conceptually distinct and well defined [20]. More recent work conceptualizes parental mediation as two higher-order strategies; enabling and restrictive, derived from previous multiple mediation practices [4]. Enabling mediation, which combines active mediation of internet use and safety with monitoring and technical controls, is positively associated with children’s online opportunities but also with their exposure to online risks. In contrast, restrictive mediation is associated with fewer risks, but also with fewer opportunities, highlighting an inherent trade-off rather than a strategy that simultaneously maximizes opportunities while minimizing risks [4]. As a result, it has been difficult to link specific mediation strategies to clearly beneficial outcomes [21]. Qualitative research suggests that the boundaries between mediation strategies are often indistinct in practice, with parents tending to employ multiple approaches guided by their personal values and contextual considerations [19]. These difficulties have further been described as a methodological challenge, as instruments used in prior research may combine practices that are potentially effective with those that are counter-effective within the same mediation category. Based on these conclusions, Lukasvá et al. [21], in developing a new scale to measure parental mediation, conceptualized overprotective mediation as strategies in which parents overuse restrictive practices. This conceptualization separates such behaviours as a potentially ineffective or even harmful mediation strategy [21].

Mediation strategies are influenced by parental characteristics. Socioeconomic background plays a significant role: higher educational attainment correlates with more active mediation, while lower education is linked to more passive approaches [22]. Gender differences are also evident, with mothers more likely to adopt restrictive or balanced mediation, i.e. monitor and regulate the child’s digital device use focusing on enabling regular discussion of preferred digital practices, and fathers tending toward permissiveness [4, 22]. Active and engaged parenting, particularly by mothers, is associated with lower levels of cyberbullying and digital overuse [17]. Qualitative research by Lafton et al. further supports the importance of parental interest and positive attitudes in shaping children’s digital experiences [19].

Language background is another relevant factor. In multilingual families, parents’ media strategies often reflect attitudes toward heritage language and bilingualism [23]. However, external constraints such as children’s preferences, time limitations, and dominant language exposure can hinder these efforts [23]. Families from minority language backgrounds often employ more active and restrictive strategies, shaped by cultural, religious, and trauma-related factors [24].

### Parental self-efficacy

Rooted in social cognitive theory, self-efficacy refers to individuals’ belief in their ability to manage tasks and challenges [25]. It is influenced by mastery experiences, observational learning, social encouragement, and emotional states [23]. Parental self-efficacy, a specific application of this concept, denotes parents’ confidence in their parenting abilities and their capacity to positively affect their child’s development [26–28].

Prior systematic reviews indicate that higher parental self-efficacy is consistently associated with more favorable outcomes for the parent–child relationship, parental mental health, and child development [29]. More recently, Glatz et al. [27] reported consistent associations between higher parental self-efficacy and more positive parenting practices (e.g., greater support and more effective parenting strategies), with longitudinal studies frequently supporting bi-directional effects between parental self-efficacy and positive parenting. Glatz & Lippold further demonstrated that parents with lower parental self-efficacy engage more frequently in online information seeking, using digital resources as a form of support and validation, but are more vulnerable to information overload, particularly when relying on informal or mixed sources [30]. Importantly, information overload was linked to source selection rather than search frequency, with exclusive use of institutional websites associated with lower overload. These findings suggest that parents’ ability to evaluate and manage the digital information environment may play a critical role in sustaining higher parental self-efficacy. Nevertheless, despite the significant role of digital platforms in modern time parenting, the relationship between parental self-efficacy and digital mediation remains insufficiently explored [30].

### Social network

Previous research indicates that parents who perceive their social network as limited report lower confidence in their own parenting abilities, whereas those who perceive their social network as rich report higher levels of parenting confidence [31]. A strong social network may also enable parents to cope more effectively with parenting challenges and reduce psychosocial stress [32] and research suggests that parents’ awareness of having access to a social network can contribute to reduced stress levels in parenting [33]. According to the Family Law and Parental Support Authority (MFOF) [11], parents’ social networks and access to support from close relatives are of substantial importance in shaping their perceptions of their parenting role and may function as a fundamental protective factor. Consistently, parents with strong support networks report greater confidence in their parenting abilities than those with few or no such supportive networks [11].

### Rationale

Increased use of digital tools among children presents new challenges for parents, amplifying the need for supporting parents in effective digital mediation [11]. However, many parents remain uncertain about how to manage these demands effectively [7]. While previous studies have examined mediation strategies and their socioeconomic correlates [12, 14, 20], research gaps persist regarding how individual characteristics (e.g. native language, and parental self-efficacy) and social-contextual characteristics (e.g. social networks) influence digital mediation practices.

The aim of this study was to investigate the association between parental individual- and social characteristics, including self-efficacy, gender, native language and social networks, and digital mediation strategies among parents of preschool and elementary school children in Sweden.

## Methods

### Population

This study is part of an ongoing evaluation of a universal parenting support programme conducted by the University of Skövde in collaboration with a small municipality in West Sweden. The current study employed a cross-sectional design, based on existing baseline survey data collected within the evaluation project. The study population comprised parents of children aged 3 to 14 years residing in the municipality. Of the total population of 32,580 inhabitants [34], approximately 4,500 individuals were identified as part of the target parent population. A convenience sampling approach was used, as the survey was distributed to all parents with children aged 3, 6, 9, 12, and 14 years, and participants self-selected into the study by choosing to respond to the survey. Inclusion criteria required that respondents had children in the specified age group, were residents in the municipality, understood written Swedish language and completed the survey during the autumn of 2024.

The study protocol was designed in accordance with the Declaration of Helsinki and was approved by the Swedish Ethical Review Authority (Ref: 2023-07555-01). Information about the study was provided to all parents before data collection and parents consented to participation by submitting the questionnaire.

### Data collection

Data was collected in autumn 2024. The survey (Appendix 1. Questionnaire) was distributed to parents of school-aged children (9, 12 and 14 years) during August 19th to September 1st and to parents of preschool-aged children (3 and 6 years) during September 9th to 22nd. The survey was made available via *Unikum*, a digital platform for educational communication and consisted of four main sections. Participants received an initial invitation to complete the survey, followed by a reminder approximately one week later. To increase response rates, a reminder post was also published on the municipality’s official Facebook page.

An a priori power calculation was conducted using within-group change in parental self-efficacy (TOPSE), as TOPSE is the only included instrument previously used in a similar context in Sweden. Based on Enebrink et al. [35], an expected mean change of 2.4 (SD 0.88) units was assumed. With a two-sided α = 0.05 and 80% power, a minimum of 158 participants was required to detect a significant pre–post change.

**Fig. 1:**
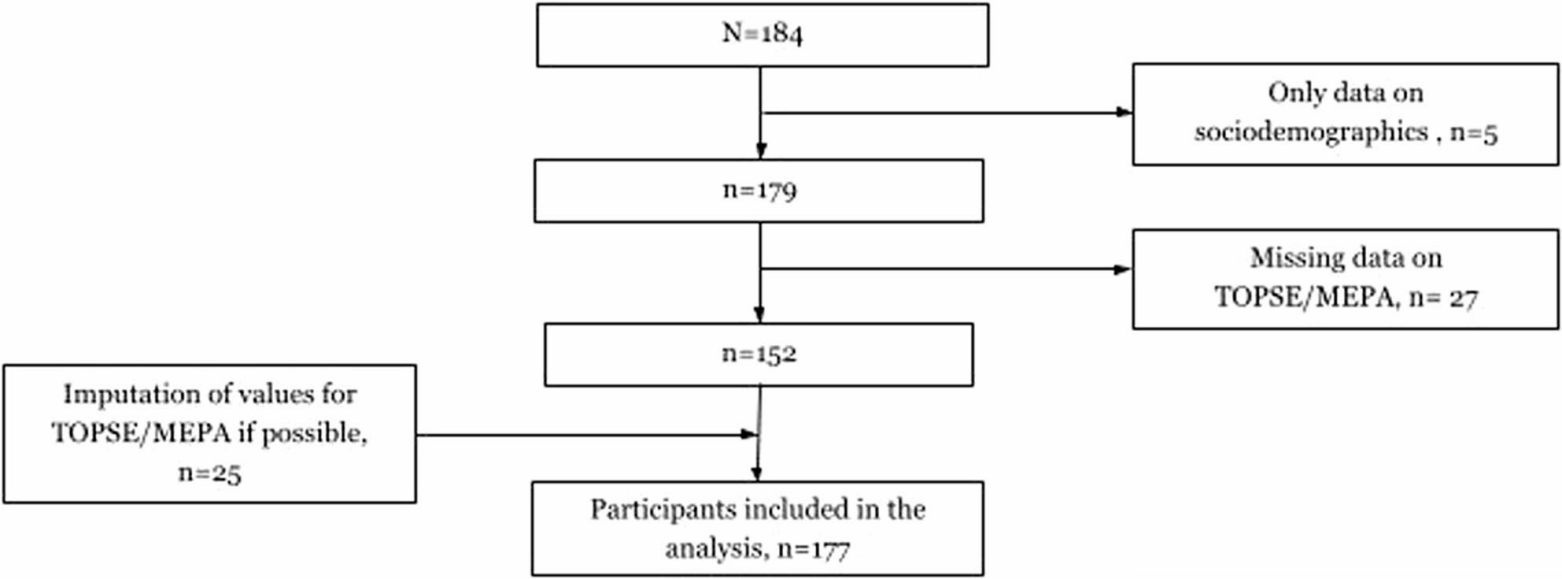
Flowchart of participant inclusion and exclusion

Of all parents’ who completed the questionnaire (*n* = 184) a total of 177 parents (96%) were eligible for analysis (Fig. 1). Five participants provided only demographic information and were removed during the initial screening. Further, single-item non-response on TOPSE/MEPA-20 instruments was observed in 25 cases. These omissions were deemed unlikely to materially affect the results; therefore, the cases were retained. For analyses requiring complete data, missing values were imputed using the gender-specific mean [36] to enable inclusion while minimising bias in the TOPSE indices and MEPA-20 outcome measures. In the end, two participants were excluded from the statistical analyses due to missing responses on multiple key items in the TOPSE and MEPA-20 instruments. No systematic patterns were detected in the missing data.

### Measures

#### Parental mediation strategies

Parental mediation strategies related to children’s screen use were assessed using the validated *Media Parenting Scale for School-Aged Children* (MEPA-20) [21, 37, 38]. The instrument comprises 20 items across three subscales: *active mediation*,* restrictive mediation*, and *overprotective mediation*. Each item is rated on a five-point Likert scale from *totally untrue* (1) to *totally true* (5).

Subscale scores were calculated as the mean of specified items: active mediation (items MP1–MP4, MP6–MP9), restrictive mediation (items MP11–MP14, MP16–MP19), and overprotective mediation (items MP5, MP10, MP15, MP20). Each sub-scale ranging from 1 to 5 points. Given the refinement of the subscales in the development of MEPA, active and restrictive mediation are considered beneficial approaches (i.e. enabling mediation strategies), as they reflect communication and boundary-setting regarding screen use including items such as; *I chat with my child about time that s/he spends using screens*, *I explain to my child which content is suitable for her/him (e.g.*,* which videos*,* games*,* apps*,* websites*,* texts*,* and pictures)*,* I chat with my child about how s/he uses screens (e.g.*,* before going to bed*,* during meals*,* and during studying)*,* I notice in which situations my child watches and uses screens*. Overprotective mediation (in some previous research referred to as restrictive mediation [22]) is viewed as a potential risk factor, as it may involve excessive control and reduced respect for the child’s autonomy and development and includes items such as; *I secretly check my child’s screen activities (when and what my child was watching or what apps s/he was using)*,* I constantly check my child’s screen activities* [21], .

The MEPA-20 has demonstrated good reliability, with acceptable-to-high internal consistency across subscales and high test–retest stability [21, 37]. Its validity is supported by a stable three-factor structure (active, restrictive, and overprotective mediation) and documented content validity in previous psychometric evaluations [21, 37]. The scale has also been successfully translated and validated in Turkish [38]. In the present study, the MEPA-20 was translated into Swedish independently by two researchers, reconciled through consensus to ensure content integrity, and subsequently reviewed by a Swedish language lecturer to ensure linguistic accuracy while preserving the meaning of all items. To assess comprehensibility and interpretation of the items, the Swedish version was pilot-tested in a group of parents not participating in the original study (*n* = 6), comprising two men and four women with children in both preschool and school. The Swedish translation of the MEPA-20 and its subscales demonstrated acceptable internal consistency reliability, with Cronbach’s α of 0.80 for active mediation, 0.87 for restrictive mediation, and 0.67 for overprotective mediation. These values were slightly higher than those reported for the original scale [21] and slightly lower than those reported for the Turkish translation [38].

#### Parental self-efficacy

Parental self-efficacy was assessed using the validated *Tool to Measure Parenting Self-Efficacy* (TOPSE) [39]. The instrument comprises 48 items across eight domains: positive emotion, being with your child, empathy, guiding, rules, pressures, acceptance, and experience. Each domain includes six items rated on an 11-point Likert scale from *completely disagree* (0) to *completely agree* [10], yielding a possible total score of 0–480 points. Higher scores reflect greater parental self-efficacy and the use of effective parenting strategies.

TOPSE is specifically designed to evaluate the impact of parenting programmes and has been widely applied in international research. The instrument has demonstrated good reliability and validity in previous psychometric evaluations, including high internal consistency (total scale α ≈ 0.94–0.95) and acceptable test–retest reliability [39]. A Swedish version of TOPSE has been translated and validated, showing good internal consistency in parents of children aged 1–14 years (total α = 0.91; subscales α = 0.66–0.83), supporting its suitability for the present study population (parents of children aged 3–14 years) [35, 40].

#### Sociodemographic characteristics and social network

Sociodemographic variables included the parent’s age (in years), gender (women/men/other), highest level of education (primary or secondary education; post-secondary education < 3 years; post-secondary education ≥ 3 years; post-secondary education ≥ 5 years), number of children (one; two; three or more), and whether Swedish was the parent’s native language (yes/no).

Parents’ social network was assessed using the question: *“Who do you talk to about your children?”* Multiple response options included: friends; social media; voluntary sector; family and relatives; neighbours; co-workers; school/preschool/school health/child health care centre; support groups; parents in the school/class; and no one. Responses were categorised into four sub-categories: *close contacts* (family and friends); *other personal contacts* (neighbours, co-workers, parents in the school/class, voluntary sector); *institutional contacts* (school/preschool/school health/child health care centre, support groups); and online forums. A participant was coded as engaging with a given category if they reported at least one of the corresponding response options. The response option “no one” was excluded from the analysis, as it was selected by only one parent. This participant was retained in the sample but was coded as not engaging in any other category.

### Statistical analysis

This cross-sectional study was analysed using IBM SPSS Statistics, version 29. Continuous variables were summarized as means (M) and standard deviations (sd) or as medians (Md) and interquartile ranges (IQR), depending on distribution. Ordinal and binary variables were presented as absolute and relative frequencies. Normality and homogeneity of variance were assessed prior to analysis. The outcome variables—active, restrictive, and overprotective mediation—were not normally distributed and, being ordinal in nature, were analysed using non-parametric methods [41]. Levene’s test indicated that the assumption of homogeneity of variance was met [41].

To identify relevant independent variables, a systematic preselection of individual and social characteristics was conducted. A broader confidence level of 80% was applied to reduce the risk of excluding variables with potential importance for subsequent multivariate analyses. The age of the parent, education and number of children in the household showed no significant associations with digital mediation strategies and were therefore excluded. Gender of the parent, native language, and all sub-categories of social network were retained as independent variables.

Associations between parental mediation strategies and individual characteristics (parental gender and native language) and sub-groups of social network were examined using Mann–Whitney U tests. Correlations between mediation strategies and parental self-efficacy (TOPSE) were assessed with Spearman’s rho. Due to its skewed distribution the parental mediation strategies were re-coded into categories using median-split (less/more mediation) and bivariate logistic regression analyses were then performed to evaluate associations between mediation strategies and parental self-efficacy, gender of the parent, native language, and sub-categories of social network. For parental self-efficacy, the variable was scaled in steps of 10 points (instead of 1) to facilitate interpretation of odds ratios, as the scale ranged from 0 to 480 points. Variables significant at *p* < 0.05 were entered into multivariate logistic regression models.

For all analyses, the significance level was set at *p* < 0.05. The analytic sample size varied slightly across analyses due to missing values in exposures and covariates.

## Results

### Descriptive statistics

Table 1 presents descriptive statistics on demographic data, native language, number of children and sub-categories of social network. A total of 177 parents answered the questionnaire, of which 76.3% were women (*n* = 135) and 23.7% were men (*n* = 42). The study population had an average age of 42 years (sd 6.8, 24–63 years). The average age was slightly higher among men (45.6 years, sd 7.5) compared to women (40.9 years, sd 6.2). Of the collected responses, 41.6% were from parents with children in preschools and 58.4% from parents with children in primary schools. For educational level, 50.8% reported a post-secondary education ≥ 3 years and 76.6% of the parents reported Swedish as their native language. Regarding the number of children in the household, 62.4% responded that they have 2 children at home. Regarding the sub-categories of social networks, participants could select multiple response options. Most reported relying on close personal contacts 97.2%) and other close contacts (63.3%), followed by institutions (57.6%), while few reported using online forums (9.0%).

**Table 1:** Descriptive statistics of the participants included in the study

Variable	Women (*n* = 135)	Men (*n* = 42)	Total (*n* = 177)
	Mean (sd)		
Age	40.9 (6.2)	45.6 (7.5)	42.0 (6.8)
	N (%)		
Highest education level			
Primary and secondary education	31 (23.0)	14 (35.0)	45 (25.7)
Post-secondary education < 3 years	31 (23.0)	10 (25.0)	41 (23.4)
Post-secondary education > 3 years	45 (33.3)	10 (25.0)	55 (31.4)
Post-secondary education > 5 years	28 (20.7)	6 (15.0)	34 (19.4)
Swedish as native language (yes)	104 (78.8)	30 (71.4%)	134 (76.6)
Number of children			
1 child	25 (18.4)	9 (21.4)	34 (19.1)
2 children	85 (62.5)	26 (61.9)	111 (62.4)
> 3 children	26 (19.1)	7 (16.7)	33 (18.5)
Data collected in			
Preschool	56 (41.2)	18 (42.9)	74 (41.6)
Primary school	79 (58.8)	24 (57.1)	103 (58.4)
Social network			
Close personal contacts	130 (96.3)	41 (100.0)	172 (97.2)
Other close contacts	87 (64.4)	25 (61.0)	112 (63.3)
Institutions	81 (60.0)	21 (51.2)	102 (57.6)
Online forums	15 (11.1)	1 (2.4)	16 (9.0)
	Median (IQR)		
TOPSE	408.0 (358.5–437.0)	384.7 (359.8-415.8)	403.0 (360.3-433.8)
Digital mediation strategies*			
Active mediation	4.38 (3.63–4.75)	4.00 (3.53–4.47)	4.25 (3.62–4.75)
Restrictive mediation	4.00 (3.25–4.63)	3.88 (3.31–4.31	4.00 (3.31–4.50)
Overprotective mediation	2.50 (1.75–3.50)	2.25 (1.75–3.25)	2.50 (1.75–3.38)

*TOPSE* Tool to Measure Parenting Self-Efficacy

*Media Parenting Scale for School-Aged Children (MEPA-20)

### Parental self-efficacy

Spearman’s rho correlation analyses were conducted to examine the relationship between parental self-efficacy (TOPSE) and the three types of parental digital mediation: active, restrictive and overprotective. The results indicated a significant positive association between parental self-efficacy and all three digital mediation strategies (Table 2). The strongest correlation was found between TOPSE and active mediation (*r* = 0.451), followed by restrictive mediation (*r* = 0.413), and overprotective mediation (*r* = 0.305). All results were statistically significant at *p* < 0.001.

**Table 2:** Spearman’s rho correlation for the relation between digital mediation strategies and parental self-efficacy

	Active mediation	Restrictive mediation	Overprotective mediation	TOPSE
Active mediation (*n* = 176)				
Restrictive mediation (*n* = 177)	0.715*			
Overprotective mediation (*n* = 177)	0.618*	0.645*		
TOPSE (*n* = 178)	0.451*	0.413*	0.305*	

*TOPSE*  Tool to Measure Parenting Self-Efficacy

*p < 0.001

### Individual characteristics of the parent

Differences in parents’ use of digital mediation strategies (active, restrictive, and overprotective) were analysed by gender and native language using Mann–Whitney U tests.

#### Gender differences

A statistically significant difference was identified between men and women for active mediation (*r* = 0.153, *p* = 0.043), with a median of 4.00 for men and 4.38 for women (Table 3). However, there were no statistically significant differences between genders regarding restrictive (*r* = 0.088, *p* = 0.247) or overprotective (*r* = 0.058, *p* = 0.444) digital mediation. Median values were similar between groups: for restrictive, the median was 3.88 for men and 4.00 for women, and for overprotective mediation 2.25 for men and 2.50 for women.

**Table 3:** Mann-Whitney U-test for differences between digital mediation strategies and gender

Digital mediation strategy	Gender	*N*	Median	U	z-value	*r*	*p*-value
Active mediation	Men	40	4.00	2133.000	-2.023	0.153	0.043
	Women	135	4.38				
Restrictive mediation	Men	41	3.88	2437.000	-1.159	0.088	0.247
	Women	135	4.00				
Overprotective mediation	Men	41	2.25	2549.500	-0.765	0.058	0.444
	Women	135	2.50				

*U* sum of rank, *z-value* standardized test statistic, *r* effect size

Significance level = *p* < 0.05

#### Swedish as native language

Across all three mediation strategies, significant differences in median values emerged between participants with Swedish as their native language and those with another linguistic background (Table 4). The median value for active mediation was 4.25 among participants with Swedish as their native language, and 4.50 among those with another native speaking background. This was statistically significant with a small effect size (*r* = -0.237, *p* = 0.002). Restrictive mediation showed a median of 3.88 among participants who had Swedish as native language, compared to 4.38 among those with another native speaking background (*r* = -0.254, *p* < 0.001). Participants with a non-Swedish native language reported higher levels of overprotective mediation (median = 3.50) compared to those with Swedish as their native language (median = 2.25), a statistically significant difference with a medium effect size (*r* = -0.402, *p* < 0.001).

**Table 4:** Mann-Whitney U-test for differences between digital mediation strategies and native language

Digital mediation strategy	Native language	*N*	Median	U	z-value	*r*	*p*-value
Active mediation	Swedish	133	4.25	1747.500	-3.106	-0.237	0.002
	Other	39	4.50				
Restrictive mediation	Swedish	134	3.88	1696.500	-3.335	-0.254	< 0.001
	Other	39	4.38				
Overprotective mediation	Swedish	134	2.25	1164.500	-5.278	-0.402	< 0.001
	Other	39	3.50				

*U* sum of rank, *z-value* standardized test statistic, *r* effect size

Significance level = *p* < 0.05

### Social network

Differences in active, restrictive, and overprotective digital mediation strategies were analysed in relation to social network variables, including personal and close contacts, institutions, and internet forums (Table 5).

**Table 5:** Mann-Whitney U-test for differences between digital mediation strategies and sub-categories of social network

Digital mediation strategy	Social network	*N*	Median	U	z-value	*r*	*p*-value
	Close personal contacts						
Active mediation	No	5	3.38	89.000	-0.946	-0.072	0.364
	Yes	169	4.25				
Restrictive mediation	No	5	2.63	101.000	-0.643	0.048	0.542
	Yes	171	4.00				
Overprotective mediation	No	5	1.75	96.000	-0.428	0.032	0.449
	Yes	171	2.50				
	Other close contacts						
Active mediation	No	65	4.25	3530.000	-0.039	-0.003	0.969
	Yes	109	4.25				
Restrictive mediation	No	65	4.00	3601.000	-0.020	-0.002	0.984
	Yes	111	3.88				
Overprotective mediation	No	65	2.50	3588.500	-0.058	-0.004	0.953
	Yes	111	2.50				
	Institutions						
Active mediation	No	72	4.13	2864.500	-2.477	-0.189	0.013
	Yes	102	4.38				
Restrictive mediation	No	74	3.75	3108.500	-1.998	-0.151	0.046
	Yes	102	4.06				
Overprotective mediation	No	74	2.00	3071.500	-2.114	-0.159	0.035
	Yes	102	2.75				
	Online forums						
Active mediation	No	158	4.25	881.500	-2.000	-0.152	0.045
	Yes	16	4.69				
Restrictive mediation	No	160	4.00	908.500	-0.055	-0.004	0.055
	Yes	16	4.50				
Overprotective mediation	No	160	2.50	907.000	-1.926	-0.145	0.054
	Yes	16	2.88				

*U* sum of rank, *z-value* standardized test statistic, *r* effect size

Significance level = *p* < 0.05

No statistically significant associations were found between *close personal contacts* for active (*r* = − 0.072, *p* = 0.364), restrictive (*r* = 0.048, *p* = 0.542), or overprotective mediation (*r* = 0.032, *p* = 0.449). Similarly, no significant associations were observed for *other close contacts*: active (*r* = − 0.003, *p* = 0.969), restrictive (*r* = − 0.002, *p* = 0.984), or overprotective mediation (*r* = − 0.004, *p* = 0.953).

In contrast, significant differences emerged for parents with *institutional network* contacts across all three mediation strategies. Parents with *institutional contacts* reported higher median scores compared to those without; active mediation (4.38 vs. 4.13, *r* = − 0.189, *p* = 0.013), restrictive mediation (4.06 vs. 3.75, *r* = − 0.151, *p* = 0.046), and overprotective mediation (2.75 vs. 2.00, *r* = − 0.159, *p* = 0.035).

*Online forum* use showed a mixed pattern. For active mediation, forum users reported a higher median score (4.69) compared to non-users (4.25), a statistically significant difference with a small effect size (*r* = − 0.152, *p* = 0.045). For restrictive mediation, the median was lower among forum users (4.00) than non-users (4.50), though this difference did not reach significance (*r* = − 0.004, *p* = 0.055). No significant difference was observed for overprotective mediation, where both groups reported a median of 2.50 (*r* = − 0.145, *p* = 0.054).

### Regression analyses

Associations between parental individual- and social characteristics and parents’ mediation strategies were further examined using bivariate and multivariate logistic regression. Separate models were estimated for active, restrictive, and overprotective mediation as dependent variables, with parental self-efficacy, gender, native language and sub-categories of social network entered as independent variables.

#### Active mediation

In the bivariate analysis, higher parental self-efficacy (TOPSE) was positively associated with more active mediation (OR 1.2, 95% CI 1.1–1.3, *p* < 0.001) (Table 6). Moreover, gender (women) was associated with more active mediation (OR 2.7, 95% CI 1.3–5.7, *p* = 0.011), as well as relying on support from *Institutions* (OR 2.1, 95% CI 1.1–4.0, *p* = 0.018). In the multivariate analysis, TOPSE remained a significant predictor (aOR 1.2, 95% CI 1.1–1.3, *p* < 0.001). The effect of gender (women) was somewhat attenuated but remained significant (aOR 2.6, 95% CI 1.1–6.0, *p* = 0.023) together with relying on *Institutions* as social network (aOR 2.2, 95% CI 1.1–4.3, *p* = 0.024).

**Table 6:** Associations between independent variables and more active mediation: results from logistic regression analyses

Variable	Bivariate analysis		
	*n*	OR (95% CI)	*p*-value
TOPSE^(increase of 10 points)	174	1.2 (1.1–1.3)***	**< 0.001**
Gender (women)	175	2.7 (1.3–5.7)*	**0.011**
Swedish as native language (no)	172	2.1 (1.0-4.3)	0.051
Social network	174	3.9 (0.4–35.3)	0.231
*Close personal contacts*			
Social network	174	0.9 (0.5–1.7)	0.846
*Other close contacts*			
Social network	174	2.1 (1.1-4.0)*	**0.018 **
*Institutions*			
Social network	174	2.6 (0.9–7.7)	0.094
*Internetforum*			
	**Multivariate analysis**		
Variable	*n* = 171	**aOR (95% CI)**	***p*** **-value**
TOPSE^(increase of 10 points)		1.2 (1.1–1.3)***	**< 0.001**
Gender (women)		2.6 (1.1-6.0)*	**0.023**
Social network		2.2 (1.1–4.3)*	**0.024**
*Institutions*			

*OR* Odds ratio, *aOR* adjusted Odds ratio, *CI* Confidence interval

^Tool to Measure Parenting Self-Efficacy (TOPSE) range from 0 to 480 points

Variables significant at *p* < 0.05 in bivariate models were entered into the multivariate model

*p < 0.05, **p < 0.01, ***p < 0.001

#### Restrictive mediation

The bivariate analysis showed that higher TOPSE scores was associated with more restrictive mediation (OR 1.2, 95% CI 1.1–1.3, *p* < 0.001) (Table 7). Moreover, not having Swedish as native language was associated with more restrictive mediation (OR 3.2, 95% CI 1.5–6.7, *p* = 0.003). In the multivariate analysis, TOPSE remained a significant predictor (aOR 1.2, 95% CI 1.1–1.2, *p* < 0.001) as well as language were parents without Swedish as native language being more likely to report more restrictive mediation (aOR 2.6, 95% CI 1.2–5.8, *p* = 0.015).

**Table 7:** Associations between independent variables and more restrictive mediation: results from logistic regression analyses

Variable	Bivariate analysis		
	*n*	OR (95% CI)	*p*-value
TOPSE^(increase of 10 points)	175	1.2 (1.1–1.3)***	**< 0.001**
Gender (women)	176	1.4 (0.7–2.9)	0.346
Swedish as native language (no)	173	3.2 (1.5–6.7)**	**0.003**
Social network	176	1.3 (0.2–7.7)	0.804
*Close personal contacts*			
Social network	176	0.9 (0.5–1.6)	0.648
*Other close contacts*			
Social network	176	1.6 (0.8–2.8)	0.156
*Institutions*			
Social network	176	2.1 (0.7–6.2)	0.158
*Internetforum*			
	**Multivariate analysis**		
Variable	*n* = 171	**OR (95% CI)**	***p*** **-value**
TOPSE^(increase of 10 points)		1.2 (1.1–1.2)***	**< 0.001**
Swedish as native language (no)		2.6 (1.2–5.8)*	**0.015**

*OR*  Odds ratio, *CI* Confidence interval

^Tool to Measure Parenting Self-Efficacy (TOPSE) range from 0 to 480 points

Variables significant at *p* < 0.05 in bivariate models were entered into the multivariate model

*p < 0.05, **p < 0.01, ***p < 0.001

#### Overprotective mediation

In the bivariate analysis, higher TOPSE scores was associated with more overprotective mediation although the 95% CI included 1.0 (OR 1.1, 95% CI 1.0–1.2, *p* = 0.004) (Table 8). Further, not having Swedish as native language was strongly associated with more overprotective mediation (OR 6.2, 95% CI 2.7–14.1, *p* < 0.001), as well as using/relying on support from *Institutions* (OR 2.4, 95% CI 1.3–4.5, *p* = 0.005). In the multivariate analysis, parents without Swedish as native language were significantly more likely to report more overprotective mediation (aOR 5.7, 95% CI 2.4–13.6, *p* < 0.001), along with relying on *Institutions* as social network (aOR 2.6, 95% CI 1.3–5.2, *p* = 0.007). TOPSE remained a statistically significant predictor of overprotective mediation, although the association was weaker than in the unadjusted model. The 95% CI for TOPSE included the null value (aOR 1.1, 95% CI 1.0–1.2, *p* = 0.037), suggesting a more uncertain effect despite statistical significance.

**Table 8:** Associations between independent variables and more overprotective mediation: results from logistic regression analyses

Variable	Bivariate analysis		
	*n*	OR (95% CI)	*p*-value
TOPSE^(increase of 10 points)	175	1.1 (1.0-1.2)**	**0.004**
Gender (women)	176	1.1 (0.5–2.1)	0.885
Swedish as native language (no)	173	6.2 (2.7–14.1)***	**< 0.001**
Social network	176	1.2 (0.2–7.5)	0.824
*Close personal contacts*			
Social network	176	1.1 (0.6–2.1)	0.712
*Other close contacts*			
Social network	176	2.4 (1.3–4.5)**	**0.005**
*Institutions*			
Social network	176	2.2 (0.8–6.3)	0.145
*Internetforum*			
	**Multivariate analysis**		
Variable	*n* = 170	**OR (95% CI)**	***p*** **-value**
TOPSE^(increase of 10 points)		1.1 (1.0-1.2)*	**0.037**
Swedish as native language (no)		5.7 (2.4–13.6)***	**< 0.001**
Social network *Institutions*		2.6 (1.3–5.2)**	**0.007**

*OR* Odds ratio, *CI* Confidence interval

^Tool to Measure Parenting Self-Efficacy (TOPSE) range from 0 to 480 points

Variables significant at *p* < 0.05 in bivariate models were entered into the multivariate model

*p < 0.05, **p < 0.01, ***p < 0.001

## Discussion

This study aimed to investigate parental individual- and social characteristics associated with digital mediation strategies, focusing on parents of children in preschool and primary school in Sweden. Our findings indicate that parental self-efficacy is consistently associated with higher levels of active and restrictive mediation. Differences were also observed by parental characteristics, with women reporting higher active mediation and parents with a non-Swedish native language reporting higher restrictive and overprotective mediation. Regarding social characteristics, institutional contacts were associated with higher levels of both active and overprotective mediation strategies.

### Parental self-efficacy and digital mediation strategies

Active digital mediation is generally regarded as a protective factor in children’s digital environments. The identified associations in this study between parental self-efficacy and both active and restrictive mediation suggest that parents with higher parental self-efficacy are more likely to engage in what previous research referred to as enabling behaviours [4] regarding children’s digital experiences. Together active and restrictive mediation represent a protective approach that may mitigate digital risks for children [21]. Our findings support the idea that mediation strategies are not mutually exclusive but may be combined depending on the situation [19]. Hence, parents with high parental self-efficacy may both promote and limit digital engagement based on what they perceive to be most beneficial for their child’s development [27] and they favour dialogue over control [14].

Regarding overprotective mediation, our results showed a significant positive association with parental self-efficacy, although the strength of the association was attenuated in the adjusted analysis and the 95% CI included 1.0 which implies that the association is uncertain. Overprotective mediation is generally regarded as a less adaptive strategy for both parents and children and may reflect parental anxiety, uncertainty, or cultural expectations rather than a sense of competence and control [30]. Elsaesser et al. have suggested that such strategies may be reactive responses to perceived risks in children’s digital environments [17]. However, restricting strategies, such as overprotective mediation, carries the risk of hindering the development of children’s digital literacy and independence [27, 29].

*Parental individual characteristics and digital mediation strategies* There was a significant gender difference in the use of active mediation strategies, with women reporting higher levels of active mediation compared to men and were more than twice as likely to adhere to more active mediation strategies independent of level of parental self-efficacy and social network. This result partially aligns with prior research suggesting that women are more likely to engage in enabling mediation strategies than men [4, 22]. One potential explanation is that mothers often take on a more prominent role in everyday parenting, including digital mediation, reflecting the gendered division of parenting responsibilities [16]. This is consistent with the findings of Nagy et al. and Lafton et al., who found that mothers engage more in communicative forms of mediation, while fathers tend to favour technical or restrictive approaches [19, 22]. No significant gender differences were observed for restrictive or overprotective mediation, which may indicate that these strategies are applied relatively evenly between genders or that other factors may be influencing the observed outcomes.

Previous studies have highlighted that factors such as mental health challenges, cultural norms, socioeconomic conditions, and language barriers [23, 29, 42] may contribute to parents’ tendency to adopt restrictive or overprotective approaches. Our present study confirms significant differences in relation to parents’ native language and their use of digital mediation strategies. Parents with other native languages than Swedish reported higher use of all three mediation strategies compared to parents with Swedish as their native language. Further, parents with other native languages than Swedish were more than twice as likely to adhere to more restrictive mediation strategies and nearly six times as likely to adhere to more overprotective mediation strategies independent of level of parental self-efficacy and social network. Previous studies including parents with a minority language background have often demonstrated greater involvement in guiding their children’s digital habits potentially reflecting both an effort to adapt to new societal norms and a desire to protect their children from risks in a partially unfamiliar digital environment [42–44]. Furthermore, parents with other native languages than Swedish may experience greater disparities in digital competence and access to digital resources [45] which may drive these parents toward more restrictive and overprotective approaches to safeguard their children in digital contexts ([22, 24]. While having a non-Swedish native language appears to play a meaningful role in shaping mediation practices, it is not a sole determinant, other contributing factors are likely at play.

### Parental social network and digital mediation strategies

This study further examined the association between social network and digital mediation strategies. Our findings suggest that relying on institutional contacts, such as school staff, child health services, or other professional support services, was linked to more than double the likelihood of parents engaging in more active mediation strategies, and to an even higher degree, over two-and-a-half-fold likelihood of engaging in more overprotective mediation strategies, independent of parental self-efficacy, gender, or native language. This suggests that institutional networks may play an important role in shaping parental mediation practices which is in line with Glatz and Lippold [30], who showed that institutional sources of information strengthen parents’ self-efficacy in addressing digital challenges. The potential role of Institutional contacts in shaping digital parenting remains an important topic for future research.

Surprisingly the results indicated that close personal contacts (family, friends) and other personal contacts (neighbours, colleagues) were not significantly associated with any of the mediation strategies. This suggests that parents’ digital mediation strategies are not strongly influenced by their immediate personal networks, contradicting Bandura’s self-efficacy theory, which emphasizes the proximal environment as central in shaping individual behaviours [26]. The constantly evolving nature of the digital landscape also highlights the relevance of Navarro and Tudge’s distinction between physical and virtual microsystems, suggesting that traditional personal networks may be losing influence as digital resources gain greater prominence [45].

### Strength and limitations

This study has several strengths. It was conducted in accordance with the STROBE checklist [46], which reduces the risk of incomplete reporting and strengthens validity and reproducibility. A priori power calculation confirmed that the required sample size was met, and validated instruments (TOPSE and MEPA-20) were used. Regression assumptions were largely satisfied, missing data were minimal and robustly handled, and ethical approval was obtained, all of which support the credibility of the findings.

Some limitations should be considered. The cross-sectional design precludes causal inference, meaning only associations can be established. The survey was conducted in Swedish, which may have limited participation among individuals with restricted language proficiency. Despite a reasonable match between the sample and the municipality in terms of age distribution, number of children, and proportion with a non-Swedish native language, women were substantially overrepresented. The low overall response rate and use of convenience sampling further limit generalizability and raise the possibility of selection bias. In addition, socially desirable responses cannot be ruled out given the sensitivity of parenting practices, and the treatment of summed Likert-scale scores as interval data may slightly underestimate variance. Further, a limitation of this study is the wide child age range (3–14 years), as parental mediation strategies may differ substantially across developmental stages. Although age-stratified analyses would have been preferable, subgroup sample sizes were too small to support robust analyses, and age-related heterogeneity may therefore have influenced the observed associations.

Overall, the study provides robust findings based on validated measures and careful methodological considerations, but the limitations related to design, sampling, and representativeness should be borne in mind when interpreting the results.

## Conclusion

This study underscores the pivotal role of parental self-efficacy in shaping digital mediation strategies. Enabling mediation strategies, such as active and restrictive mediation, can function as complementary protective resources suggesting that a balanced approach could be the most beneficial for children’s digital well-being. However, parents may need specific guidance to turn their parenting confidence into effective strategies for managing their children’s digital activities. The findings highlight the influence of language background, as parents with non-Swedish native language reported higher levels of restrictive and overprotective mediation. This pattern illustrates how cultural and linguistic factors shape digital parenting strategies and points to ‘cultural translation’ in parenting support, extending beyond the mere linguistic adaptation of materials, to address underlying cultural norms and values. This emphasises the importance of developing support interventions for parents that address both cultural context and confidence in their parenting abilities regarding digital mediation. The study’s findings also support Sweden’s public health goals of promoting safe, inclusive environments and equal health opportunities for children and families. These results align with Agenda 2030 targets on reducing inequalities and fostering digital inclusion, highlighting the need for supportive measures that strengthen parental self-efficacy and equitable access to digital resources.

Furthermore, the stronger influence of institutional networks compared to personal ties may signal a societal shift in how parents receive and act on information about digital parenting. This insight is particularly relevant for policymakers and public health practitioners, as it suggests that institutional actors have a unique responsibility to communicate balanced messages about digital opportunities and risks. Therefore, identifying the most effective channels for reaching parents is essential to equal access for support.

## Supplementary Information

Supplementary Material 1.

## Data Availability

The data that support the findings of this study are not publicly available due to the Swedish Ethical Review Authority regulations, but are available from the corresponding author on reasonable request.
