# Supplementary material for: Digital mediation, parental self-efficacy and social context: a cross-sectional study on parents in Sweden
Source: BMC Public Health. 2026 Apr 15;26:1246. doi: 10.1186/s12889-026-27351-y (PMC13085379; doi:10.1186/s12889-026-27351-y)
Supplement: Supplementary file 1 — Supplementary Material 1. [file 12889_2026_27351_MOESM1_ESM.pdf]

Questionnaire

I am

- o Women
- o Man
- o Other

Year of birth ______

In general, how would you rate your health?

- o Very good
- o Good
- o Fair
- o Poor
- o Very poor
- o Prefer not to say

My highest level of education is

- o Primary education
- o Upper secondary education
- o Post-secondary education < 3 years
- o Post-secondary education ≥ 3 years
- o Post-secondary education ≥ 5 years
- o Prefer not to say

Number of children in my household

- o 1 child
- o 2 children
- o 3 children
- o 4 children
- o More than 4 children
- o Prefer not to say

What grade(s) are your child(ren) currently in? (Select all that apply)

- o Preschool
- o Preschool class
- o Grade 1
- o Grade 2
- o Grade 3

- Grade 4
- Grade 5
- Grade 6
- Grade 7
- Grade 8
- Grade 9
- Has completed compulsory school
- Prefer not to say

Which area of Ale do you live in?

- Southern Ale (Surte, Bohus)
- Central Ale (Nödinge, Nol, Alafors)
- Northern Ale (Älvängen, Alvhem, Skepplanda, Starrkärr/Kilanda, Hålanda)
- Prefer not to say

Is Swedish your native language?

- Yes
- No
- Prefer not to say

Have you ever wanted support in your parenting?

- Yes
- No
- Prefer not to say

Do you know where in Ale Municipality you can go for advice, support, and help in your parenting?

- Yes
- No
- Prefer not to say

Do you know where to find information, advice, and tips online about parenting?

- Yes
- No
- Prefer not to say

Do you usually talk to or cooperate with other parents about challenges that arise?

- Yes

HÖGSKOLAN
I SKÖVDE

- No
- Prefer not to say

Who do you talk to about your children? (Select all that apply)

- Friends
- Social media
- Voluntary sector
- Family and relatives
- Neighbours
- Co-workers
- School/preschool
- School health
- Child health care centre
- Support groups
- Parents in the school/class
- No one
- Prefer not to say

HÖGSKOLAN I SKÖVDE

Questions about you as a parent

The following questions concern how you see yourself as a parent. We would appreciate it if you answered all of the statements. Please tick the number that corresponds to how strongly you agree with each statement.

The scale ranges from 0 (do not agree at all) to 10 (completely agree).

The child I am referring to when responding to the following statements is

- o 3 years old
- o 6 years old
- o 9 years old
- o 12 years old
- o 14 years old

0	1	2	3	4	5	6	7	8	9	10
do not agree at all					Partially agree					Completely agree

**Emotions and affection**	0	1	2	3	4	5	6	7	8	9	10
I can show my child affection	O	O	O	O	O	O	O	O	O	O	O
I can tell when my child is happy or sad	O	O	O	O	O	O	O	O	O	O	O
I'm confident my child will come to me if she or he is sad	O	O	O	O	O	O	O	O	O	O	O
When my child is sad, I understand why	O	O	O	O	O	O	O	O	O	O	O
I have a good relationship with my child	O	O	O	O	O	O	O	O	O	O	O
I find it hard to cuddle and snuggle with my child	O	O	O	O	O	O	O	O	O	O	O

**Play and joy**	0	1	2	3	4	5	6	7	8	9	10
I can have fun with my child	O	O	O	O	O	O	O	O	O	O	O
I can enjoy every stage of my child's development	O	O	O	O	O	O	O	O	O	O	O
I can spend cozy days with my child	O	O	O	O	O	O	O	O	O	O	O
I can plan activities that my child enjoys	O	O	O	O	O	O	O	O	O	O	O
It comes naturally to me to play with my child	O	O	O	O	O	O	O	O	O	O	O
I can help my child develop to the fullest of their potential	O	O	O	O	O	O	O	O	O	O	O

HÖGSKOLAN I SKÖVDE

**Compassion and understanding**	0	1	2	3	4	5	6	7	8	9	10
I have the patience to explain things clearly to my child	O	O	O	O	O	O	O	O	O	O	O
I can get my child to listen to me	O	O	O	O	O	O	O	O	O	O	O
I can comfort my child	O	O	O	O	O	O	O	O	O	O	O
I can listen to my child	O	O	O	O	O	O	O	O	O	O	O
I can put myself in my child's situation	O	O	O	O	O	O	O	O	O	O	O
I understand my child's needs	O	O	O	O	O	O	O	O	O	O	O

**Guidance**	0	1	2	3	4	5	6	7	8	9	10
As a parent, I feel in control	O	O	O	O	O	O	O	O	O	O	O
My child accepts the boundaries I set	O	O	O	O	O	O	O	O	O	O	O
I get my child to behave well without any arguments	O	O	O	O	O	O	O	O	O	O	O
I stay calm when I encounter problems	O	O	O	O	O	O	O	O	O	O	O
I have a hard time getting my child to stop when he/she misbehaves	O	O	O	O	O	O	O	O	O	O	O
I stay calm when my child misbehaves	O	O	O	O	O	O	O	O	O	O	O

**Setting boundaries**	0	1	2	3	4	5	6	7	8	9	10
I find it easy to set boundaries	O	O	O	O	O	O	O	O	O	O	O
I can stand by the rules I've set for my child	O	O	O	O	O	O	O	O	O	O	O
I can reason with my child	O	O	O	O	O	O	O	O	O	O	O
I can find ways to avoid conflicts	O	O	O	O	O	O	O	O	O	O	O
I am consistent in setting boundaries	O	O	O	O	O	O	O	O	O	O	O
I can set boundaries for my child without feeling guilty	O	O	O	O	O	O	O	O	O	O	O

**Other people's opinions**	0	1	2	3	4	5	6	7	8	9	10
I have a hard time dealing with other people's expectations of me as a parent	O	O	O	O	O	O	O	O	O	O	O
I can't stand up for my opinions when others tell me how to take care of my child	O	O	O	O	O	O	O	O	O	O	O

If I listen to other people's advice, it becomes hard for me to decide what to do	O	O	O	O	O	O	O	O	O	O	O
I speak up to people if I don't agree with them	O	O	O	O	O	O	O	O	O	O	O
I can ignore other people's opinions on how things should be done	O	O	O	O	O	O	O	O	O	O	O
I don't feel the need to compare myself to other parents	O	O	O	O	O	O	O	O	O	O	O

**How I see myself as a parent**	0	1	2	3	4	5	6	7	8	9	10
I know I am good enough as a parent	O	O	O	O	O	O	O	O	O	O	O
I handle the demands of parenthood just as well as other parents	O	O	O	O	O	O	O	O	O	O	O
I haven't been very successful as a parent	O	O	O	O	O	O	O	O	O	O	O
As a parent, I can handle most things without losing my composure	O	O	O	O	O	O	O	O	O	O	O
I can be there for my child	O	O	O	O	O	O	O	O	O	O	O
My child feels safe when I'm around	O	O	O	O	O	O	O	O	O	O	O

**Knowledge and experience**	0	1	2	3	4	5	6	7	8	9	10
I can recognize changes in my child's development	O	O	O	O	O	O	O	O	O	O	O
I can share experiences and ideas with other parents	O	O	O	O	O	O	O	O	O	O	O
I can learn new ways to interact with my child	O	O	O	O	O	O	O	O	O	O	O
I can make the changes needed to improve my child's behavior	O	O	O	O	O	O	O	O	O	O	O
I can handle most problems with the help of others' advice	O	O	O	O	O	O	O	O	O	O	O
Knowing that others have similar difficulties and problems with their children makes it easier for me as a parent	O	O	O	O	O	O	O	O	O	O	O

HÖGSKOLAN I SKÖVDE

Questions about the parental role and digital media

The following questions are about how you, as a parent, approach your child's use of digital media. We would appreciate it if you answered all of the statements. Please tick the number that corresponds to how strongly you agree with each statement.

The scale ranges from 1 (totally untrue) to 5 (totally true)

1	2	3	4	5
Totally untrue	Rather untrue	Neither true nor untrue	Rather true	Totally true

	1	2	3	4	5
I chat with my child about the time that s/he spends using screens	O	O	O	O	O
My child sees that I am able to regulate my screen time	O	O	O	O	O
I explain to my child which content is suitable for her/him (e.g., which videos, games, apps, websites, texts, and pictures)	O	O	O	O	O
I help my child to find suitable content (e.g., videos, games, apps, websites, texts and pictures)	O	O	O	O	O
I secretly check my child's screen activities (when and what my child was watching or what apps s/he was using)	O	O	O	O	O
In front of my child, I consume only such content (e.g., videos, games, apps, websites, texts, and pictures) that is also suitable for my child	O	O	O	O	O
I chat with my child about how s/he uses screens (e.g., before going to bed, during meals, and during studying)	O	O	O	O	O
I explain to my child in what situations the use of screens is in/appropriate	O	O	O	O	O
I help my child to find suitable situations for watching/using screens	O	O	O	O	O

I let my child know that watching/using screens is a waste of time	O	O	O	O	O
We have agreed rules about screen time	O	O	O	O	O
I do not let my child use screens longer than agreed	O	O	O	O	O
I notice what content my child consumes through screens (e.g., what videos s/he watches, games s/he plays, apps s/he uses, text, and pictures).	O	O	O	O	O
We have agreed rules about what my child may and may not watch on/do on screens (e.g., which videos, games, apps, texts, and pictures)	O	O	O	O	O
I constantly check my child's screen activities	O	O	O	O	O
I do not let my child consume other content than agreed.	O	O	O	O	O
I notice in which situations my child watches and uses screens	O	O	O	O	O
We have rules specifying situations in which my child is or is not allowed to watch/use screens	O	O	O	O	O
I do not let my child use screens in other than agreed situations	O	O	O	O	O
In our family, we set rules which almost never allow children to use screens for entertainment	O	O	O	O	O

Thank you for taking the time to answer the questionnaire!
